# TreeViewer: Flexible, modular software to visualise and manipulate phylogenetic trees

**DOI:** 10.1002/ece3.10873

**Published:** 2024-02-01

**Authors:** Giorgio Bianchini, Patricia Sánchez‐Baracaldo

**Affiliations:** ^1^ School of Geographical Sciences University of Bristol Bristol UK

**Keywords:** figures, graphical interface, Newick, NEXUS, phylogenetic trees, phylogenetics

## Abstract

Phylogenetic trees illustrate evolutionary relationships between taxa or genes. Tree figures are crucial when presenting results and data, and by creating clear and effective plots, researchers can describe many kinds of evolutionary patterns. However, producing tree plots can be a time‐consuming task, especially as multiple different programs are often needed to adjust and illustrate all data associated with a tree. We present TreeViewer, a new software to draw phylogenetic trees. TreeViewer is flexible, modular, and user‐friendly. Plots are produced as the result of a user‐defined pipeline, which can be finely customised and easily applied to different trees. Every feature of the program is documented and easily accessible, either in the online manual or within the program's interface. We show how TreeViewer can be used to produce publication‐ready figures, saving time by not requiring additional graphical post‐processing tools. TreeViewer is freely available for Windows, macOS, and Linux operating systems and distributed under an AGPLv3 licence from https://treeviewer.org. It has a graphical user interface (GUI), as well as a command‐line interface, which is useful to work with very large trees and for automated pipelines. A detailed user manual with examples and tutorials is also available. TreeViewer is mainly aimed at users wishing to produce highly customised, publication‐quality tree figures using a single GUI software tool. Compared to other GUI tools, TreeViewer offers a richer feature set and a finer degree of customisation. Compared to command‐line‐based tools and software libraries, TreeViewer's graphical interface is more accessible. The flexibility of TreeViewer's approach to phylogenetic tree plotting enables the program to produce a wide variety of publication‐ready figures. Users are encouraged to create their own custom modules to expand the functionalities of the program. This sets the scene for an ever‐expanding and ever‐adapting software framework that can easily adjust to respond to new challenges.

## Graphical abstract

TreeViewer is a new software to draw phylogenetic trees that is flexible, modular, and user‐friendly. Plots are produced as the result of a user‐defined pipeline, which can be finely customised and easily applied to different trees. TreeViewer is mainly aimed at users wishing to produce highly customised, publication‐quality tree figures using a single GUI software tool and is released under an AGPLv3 licence for Windows, Linux, and macOS operating systems.

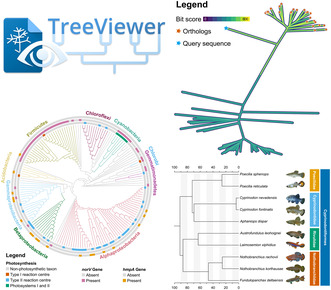

## 1 BACKGROUND

TreeViewer is a flexible and user‐friendly multiplatform software for bioinformaticians and evolutionary biologists. TreeViewer helps users to visualise phylogenetic trees with the option of displaying additional data (e.g., sequence alignments and character states) enabling them to identify novel evolutionary patterns. Complex phylogenetic tree plots can be produced by customising pipelines depending on the user's needs. These pipelines can later be reused or adapted to other projects, optimising the user's time. TreeViewer is designed with the aim of producing high‐quality publication figures and also provides a command‐line interface that can be used to work with large‐scale trees and/or to automatically produce plots as part of a script pipeline.

Tree plots produced by the program are the result of the concerted action of multiple “modules” (similar to the structure of Mesquite (Maddison & Maddison, 2023)). There are currently nine different types of modules available in TreeViewer (Table 1). Users can choose from these modules and arrange them depending on their needs, therefore customising every step of the workflow. Furthermore, this modular design means that changes to the settings of an individual module are independent of the rest of the chosen modules. As a result, changes made to a tree can be easily undone by disabling the module that is responsible for them.

**TABLE 1:** Types of modules available in TreeViewer. The table shows the task accomplished by each module, as well as an example of each type of module.

Module type	Task	Example
File type	Opens and interprets the contents of a tree file in a particular format	The “Newick” module adds support for tree files in Newick format
Load file	Loads the contents of a tree file, making them available for TreeViewer	The “Memory loader” module loads the trees in memory
Transformer	Transforms the trees contained in a tree file, producing a single tree that can be further processed	The “Consensus” module computes a consensus tree out of the trees contained in the file
Further transformation	Performs additional transformations on the tree	The “Reroot tree” module re‐roots the tree
Coordinates	Computes the coordinates of the tree nodes in the plot	The “Rectangular” module computes the coordinates for a tree in a rectangular style
Plot action	Plots an element of the tree	The “Branches” module plots the tree branches
Action	Performs a generic action, which can involve activating or de‐activating some modules	The “Rooted tree style” module changes the current *Coordinates* module and activates *Plot action* modules in order to plot the tree as a rectangular rooted tree
Selection action	Performs an action on the selected node	The “Collapse selection” module collapses the selected node (by activating the “Collapse node” *Further transformation* module)
Menu action	Adds an item to the menus in the main window	The “Export” module adds an option to export the plot as a PDF document or an SVG, PNG, or TIFF image

Plots created with TreeViewer are intrinsically *reproducible*. In addition to saving the final plot as a publication‐ready figure, users can choose to keep track of the whole pipeline that was involved in producing the plot starting from a phylogenetic tree file. This means that if a small change is needed somewhere along the pipeline, it can be applied in‐place, without having to repeat all the steps that were involved in creating the initial plot (e.g., selecting the colour for the branches and collapsing some nodes). Furthermore, it is possible to apply the same pipeline to a different tree. Users can, therefore, create their own “plot style” and readily apply it to different datasets, performing only minimal dataset‐specific adjustments. For example, a phylogenetic tree plot could be created using provisional data while a long analysis is running and then updated when the final tree becomes available, thus increasing the potential for multitasking.

Users can also create new modules in TreeViewer. A curated online repository of modules (Bianchini, 2021a) keeps users informed as to when new modules are released and/or a module is modified. The lightweight development environment integrated into TreeViewer enables advanced users to create modules that are tailored to their particular needs. These can later be shared with other users by uploading them to the TreeViewer module repository.

TreeViewer is mainly a graphical program, in which users can interact with the phylogenetic tree plot by resizing it, selecting individual nodes, and performing context‐specific actions (e.g., pruning a node or collapsing it). In the era of big data (Marx, 2013), it is nowadays common to have phylogenetic trees involving tens or hundreds of thousands of taxa (e.g., Hug et al., 2016; Naafs et al., 2021; Rabosky et al., 2018; Smith & Brown, 2018; Upham et al., 2019); this is a major challenge that makes working with large‐scale trees impractical and time‐consuming. TreeViewer has been designed with these issues in mind, and it provides a lightweight command‐line interface that can be used to manipulate and plot the trees either interactively or as part of an automated script pipeline.

**FIGURE 1:**
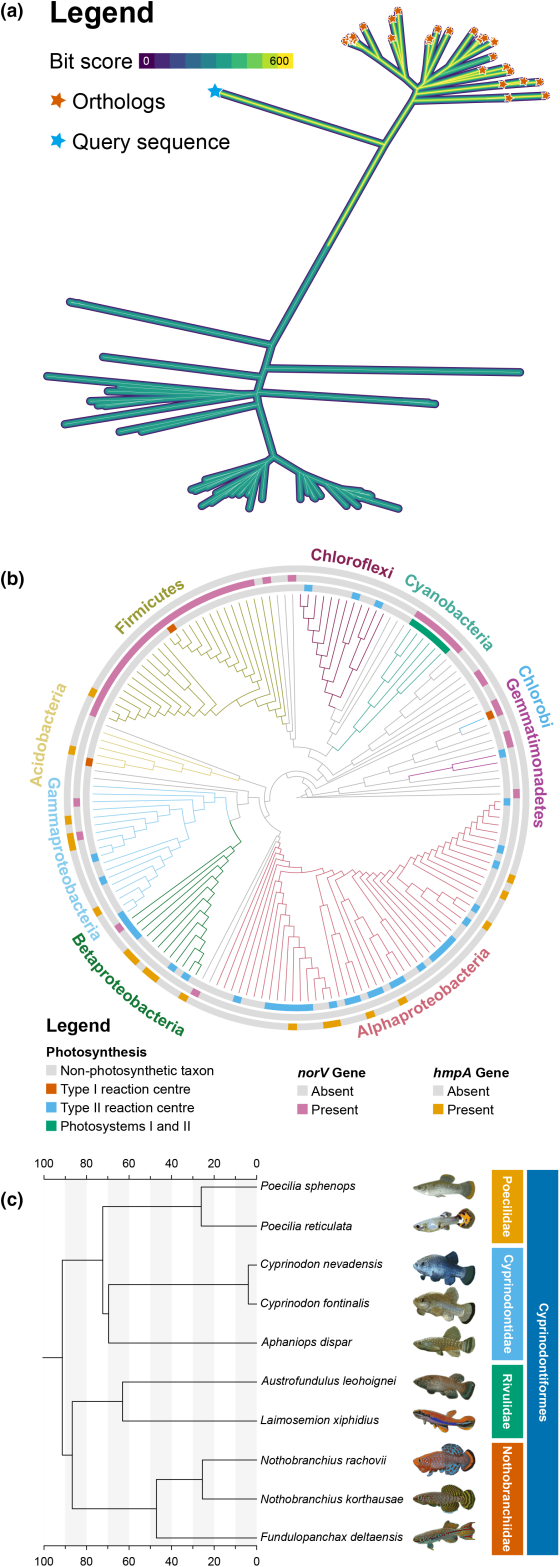
Examples of trees created using TreeViewer. (a) An unrooted tree showing the results of a BLAST (Camacho et al., 2009) search. The branch colour displays the BLAST score of each hit, and orange stars highlight the “true orthologs” of the query sequence, which is denoted by the blue star. (b) A circular tree showing the presence or absence in various bacterial strains of photosynthetic reaction centres and of the *norV* and *hmpA* genes. (c) A time‐calibrated tree of cyprinodontiform fishes. The tree includes an image for each species. Fish families are also highlighted. The phylogeny is adapted from (Rabosky et al., 2018). The images for *P. reticulata* and *P. sphenops* are adapted from original photographs (Eisfeld, 2016; Torres, 2010), while all the other images are adapted with permission from original photographs by R. Pohlmann (Pohlmann, 2016). All figures were created exclusively using TreeViewer; no other external graphics editing software was used. Instructions detailing how to create these plots (and more) are available in the TreeViewer online manual (Bianchini, 2021e).

A number of examples and tutorials make it possible for users to easily gain confidence with the program and learn to use all of its features. These are available in the online documentation (Bianchini, 2021b) and describe a large variety of different plots, some of which are shown in Figure 1. Plots created using TreeViewer have been included in recent publications (Anderegg et al., 2022; Beach et al., 2022; Gyimesi & Hediger, 2022; Iwata et al., 2023; Klau et al., 2022; Moreira et al., 2022; Naafs et al., 2021; Sánchez‐Baracaldo et al., 2022; Sasoni et al., 2022; Sobol et al., 2022; Visagie et al., 2023; Visagie & Yilmaz, 2023; Wu & Guo, 2022).

## 2 IMPLEMENTATION

TreeViewer is written using the C# programming language, running under the .NET 7 runtime. Executables are available for computers running Windows and Linux on x64 processors (e.g., Intel or AMD processors) and for macOS computers running on x64 (Intel) or arm64 (Apple Silicon) processors. As ARM‐based Windows and Linux computers become more widely adopted, arm64 versions of the program for these operating systems will be provided.

TreeViewer is an open‐source software, released under the GNU AGPLv3 licence (Free Software Foundation, 2007). In addition to the base libraries included in the .NET runtime, the program relies on a number of open‐source libraries to perform various tasks (e.g., drawing graphics, providing a GUI, computing statistics, and manipulating phylogenetic trees). The full list of libraries used by TreeViewer, along with licensing information, is available in the licence agreement that is displayed when installing the program.

### 2.1 User interface

**FIGURE 2:**
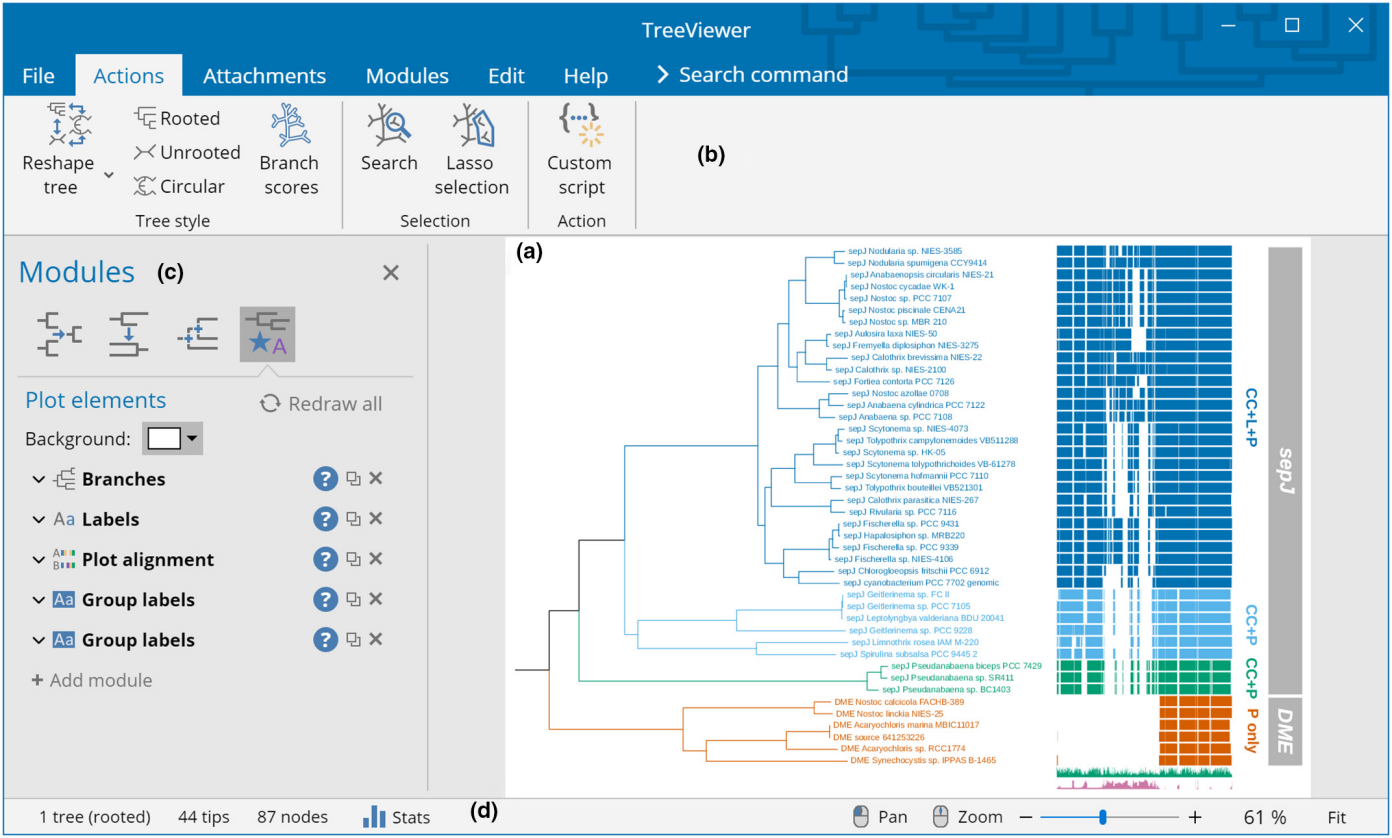
Main user interface of TreeViewer. (a) The tree plot is shown in the main part of the window. (b) Above the tree plot, a toolbar contains buttons to perform actions on the plot. (c) On the left of the tree plot, a panel shows the modules that are currently enabled, along with their settings. The blue question mark button next to each module directly opens the manual for that module. (d) The status bar at the bottom of the interface contains general information about the tree, as well as controls to determine the zoom level of the interface.

TreeViewer provides a user‐friendly graphical user interface (GUI, Figure 2). The user interface (UI) design was inspired both from other popular software to draw phylogenetic trees (e.g., FigTree; Rambaut, 2018) and from commonly used office suite software (e.g., Microsoft Office). A consistent UI across all supported platforms is achieved by using the Avalonia UI framework (AvaloniaUI Organization, 2013); this makes it possible for the main UI elements to always have the same appearance, while platform‐specific dialogs are used where appropriate (e.g., in file dialogs).

**FIGURE 3:**
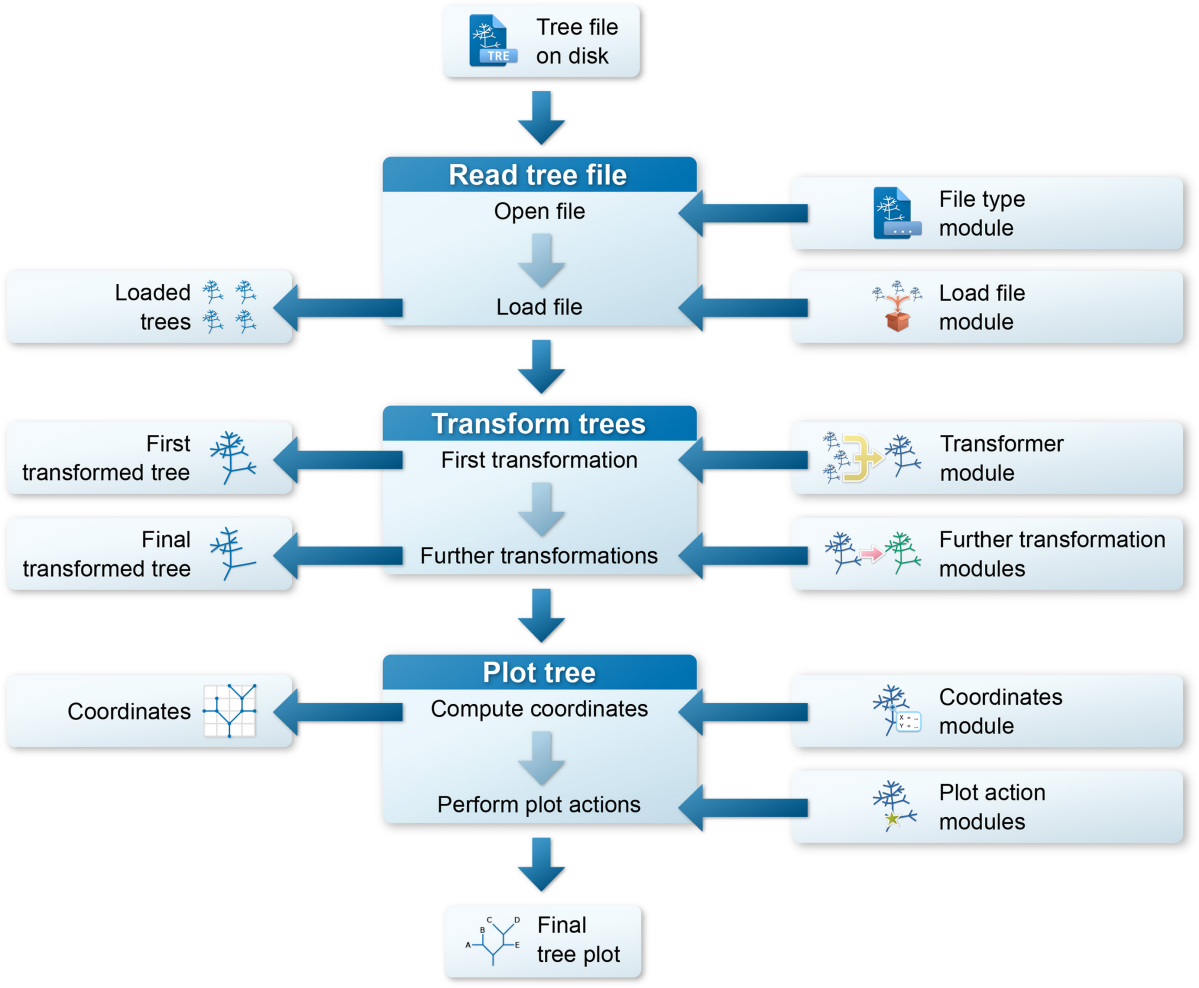
TreeViewer workflow to create tree plots. First of all, the program reads a tree file containing one or more trees using a *File type* and a *Load file* module. The tree(s) are then transformed using a *Transformer* module and, if necessary, one or more *Further transformation* modules. The final transformed tree is then plotted using a *Coordinates* module and one or more *Plot action* modules.

The interface of TreeViewer is used to explicitly describe the process that goes from a tree file to a finished tree plot (Figure 3) in a series of discrete steps. The initial step in producing a plot is reading a tree file from the hard disk. This involves opening the file, determining its format, and choosing the most appropriate module to read it. These actions are performed by a *File type* module (Table 1). The program can automatically choose the best module for each file, but users can also decide to manually specify the module they want to use, in case multiple modules are able to read the same file type. The *File type* modules that are currently available enable TreeViewer to open tree files in formats such as Newick (Felsenstein, 1986), NEXUS (Maddison et al., 1997), NCBI ASN.1 (Ostell, 1995), and a novel binary tree format (Bianchini, 2023a). TreeViewer then determines the best strategy to load in memory the tree file that has been read. This action is performed by a *Load file* module (Table 1), which can be used, for example, to copy the contents of the entire file into the system's memory (for smaller files) or to read just one tree at a time from the file as necessary (for larger files containing many trees).

Once a tree file has been read, TreeViewer has access to a list containing one or more phylogenetic trees, depending on how many were present in the tree file. If the file contains multiple trees, the next step is to produce a single tree that will then be subsequently plotted. This step is performed by a *Transformer* module (Table 1) and can involve, for example, computing a consensus tree or just choosing a single tree out of the ones that are in the file. Before plotting, the tree produced by the *Transformer* module (i.e., the “First transformed tree”) can also be further modified by one or more *Further transformation* (Table 1) modules; these modules perform actions such as re‐rooting the tree, collapsing nodes, and rotating them. Once all the *Further transformation* modules have acted, a “Final transformed tree” is produced, which is the tree that is plotted in the subsequent steps.

In order to plot a tree, the next step is determining the coordinates of the nodes in the tree, that is, at which position in the plot each branch will start and end. This step is performed by a *Coordinates* (Table 1) module, which determines, for example, whether the tree appears as an unrooted tree or as a rectangular tree. Finally, this set of coordinates is used by *Plot action* (Table 1) modules to create the phylogenetic tree plot. Each module contributes to the plot with a single feature, such as the branches of the tree, the labels for the tip nodes, or a scale bar.

The final tree plot is the result of the action of all these modules. It can be viewed and manipulated in the graphical interface of TreeViewer, which allows users to click on branches to select them, display their attributes, and perform actions on them; plots can also be exported as publication‐ready PDF, SVG, PNG, or TIFF files.

### 2.2 Command‐line interface

**FIGURE 4:**
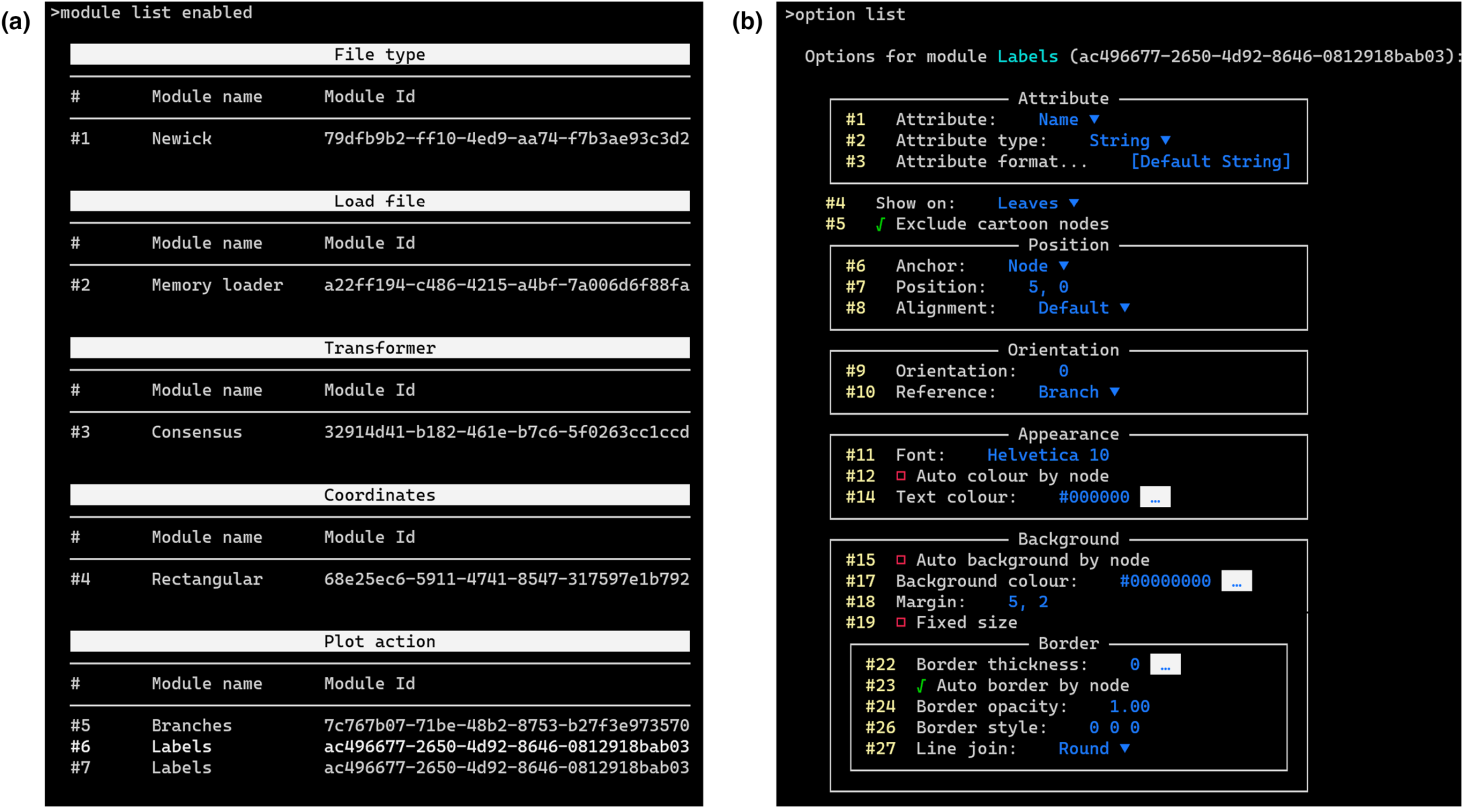
TreeViewer command‐line interface. (a) List of currently enabled modules. (b) List of options and current values for the *Labels* module. Colours are used to help users navigate the interface, distinguishing between the parameter index (yellow), the name of the parameter (white), and its value (blue). If required, the colours can be easily disabled, as TreeViewer follows the NO_COLOR standard (Stein, 2017).

In addition to the GUI, TreeViewer provides a command‐line interface (CLI, Figure 4). The TreeViewer CLI has two main use cases: the first one is to create plots for very large trees (e.g., >100,000 taxa) that may be impractical to handle using the GUI, and the second one is to be included in phylogenetic analysis pipelines. From the TreeViewer CLI, it is possible to export renderings of the tree plots (e.g., in PDF or SVG format), as well as to save tree files that contain module information. These files can then be opened with the TreeViewer GUI (e.g., to inspect attribute values at some branches in the tree or to collapse/expand nodes).

Interacting with the TreeViewer CLI is similar to interacting with the TreeViewer GUI: for example, the modules that are currently enabled can be listed, their current parameters viewed or modified, and nodes can be “selected.” The main difference is that the interaction happens through commands issued to the CLI, rather than through clicks on the GUI. The “help” command can be used to list all available commands, as well as to show more information about a particular command. Tab completion is available across the CLI, for command names, parameters, and file names (e.g., when opening or saving a file).

For pipeline integration, the commands can be stored on a file and then fed to the standard input of the TreeViewer CLI. This can be used, for example, to create a template command file with placeholders for key parameters (e.g., the name of the file to open or the accession numbers of the outgroup taxa); the pipeline would then replace the placeholders with the actual values for a certain analysis and eventually feed the resulting command file to the TreeViewer CLI.

### 2.3 Modular structure

The modular structure of the software is enabled by the self‐contained Roslyn .NET Compiler Platform (Microsoft Corporation, 2015). Modules are distributed as compressed source code archives that also contain a digital signature and metadata about the module (e.g., the name of the author, the version of the module, and a short description). After the digital signature of a module has been verified (or the user has agreed to install a module with an unverified signature), the source code of the module is compiled by TreeViewer using the Roslyn application programming interface (API). The resulting compiled assembly is then stored on the user's disk and loaded automatically when the program is opened.

This approach makes it possible to distribute identical module source files to users on any platform, because the appropriate platform‐specific artifacts are generated directly on the end‐user's machine. In principle, it is similar to the distribution systems used by many other extensible frameworks, such as R or Python packages (Hornik & Leisch, 1997; Python Software Foundation, 2003). The “Module manager” and “Module repository” windows available within the software can be used to see which modules have been installed or are available, providing access to the source code and the documentation for each module. At the time of writing, 98 modules are available for TreeViewer, which perform various tasks.

Source code compilation using the Roslyn API is not instantaneous, even though it normally does not take more than a few seconds, including the most complex modules. This time penalty is avoided by storing the compiled artifacts on the user's disk. However, once the source code has been compiled, its performance is undistinguishable from the performance of code included in the “native” TreeViewer assemblies. This allows TreeViewer to use runtime‐compiled code not only for modules, but also for many small and apparently trivial tasks.

For example, consider the operation of displaying the value of a numerical attribute (e.g., the length of a branch) on the tree. In order to do this, it is necessary to convert the attribute value from a “number” (stored, e.g., as an IEEE 754 double‐precision binary floating‐point value (Floating‐Point Working Group, 2019)) to a textual representation (e.g., “1.234”). This could be achieved in many ways. For example, users may want to show a certain number of decimal digits or significant digits; they may want to round it, truncate it towards 0, or truncate it away from zero; and they may want to show it in decimal notation or scientific notation. While some of these combinations are exposed through controls in the UI of the program, it would not be realistic to have a different button for every possibility. The *Labels* module of TreeViewer addresses this problem by exposing the source code of its “Attribute formatter,” that is, the piece of code that transforms an attribute value into a text string.

**FIGURE 5:**
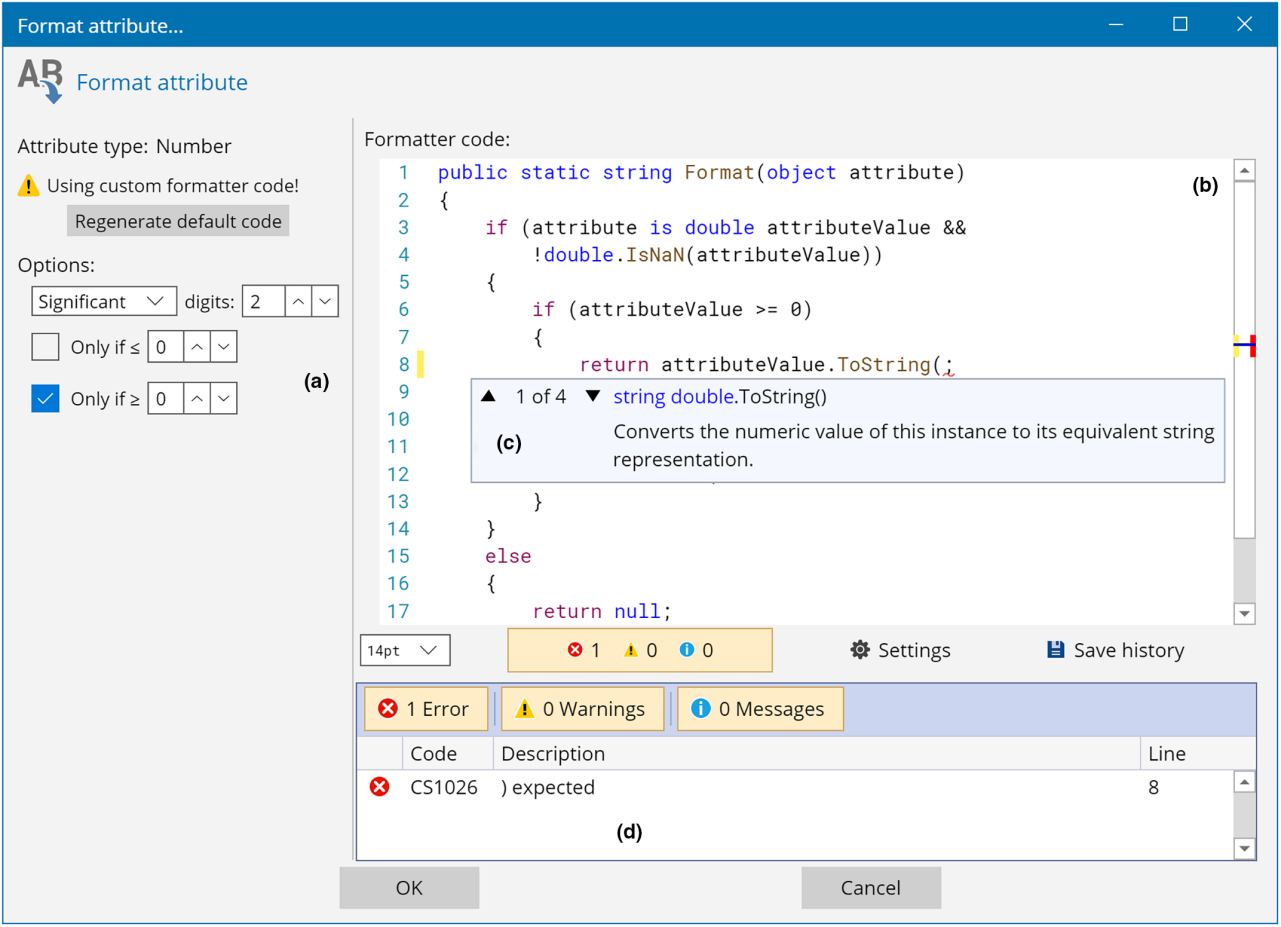
Attribute formatter window. Attribute formatters are used to convert attribute values from one type to another. In the window shown, users can convert a numerical attribute into a text string. (a) Controls to automatically generate code for simple use cases. (b) User‐modifiable source code of the method used to convert the attribute value. (c) Intelligent code completion with in‐line documentation. (d) Real‐time error checking.

The GUI (Figure 5a) contains controls to automatically generate the source code for simple cases, but the code itself (Figure 5b) can be fully customised by users according to their requirements. Furthermore, the integration between the software and the compiler makes it possible for code editor windows in TreeViewer to provide many features that make writing source code as user‐friendly as possible. These include syntax highlighting, semantic highlighting (where code tokens such as class and method names are highlighted based on their meaning), intelligent code completion with in‐line documentation (Figure 5c), real‐time error checking (Figure 5d), and breakpoints (which can be used to inspect the state of the code and the value of local variables during its execution). This is achieved through the CSharpEditor control (Bianchini, 2023b).

This approach of including UI controls that automatically generate source code for simple use cases, while allowing users to manually fine‐tune the code, is ubiquitous throughout TreeViewer. Many modules use attribute formatters to convert between attribute types (Table 2), and they can all be customised with an interface similar to Figure 5. The downside of such an approach is that, when the module settings are included in files exported by TreeViewer, each tree file becomes essentially a small program. In principle, hostile parties could include malicious code in an attribute formatter or another customisable code item and use the tree file as an infection vector. To address this issue, tree files created by TreeViewer can be signed in the same way as module files are (see Section 2.5).

**TABLE 2:** Attribute formatters. Attribute formatters are distinguished by the type of data they output. For each formatter type, the table shows the available input data types and the default conversions defined for each.

Attribute formatter type	Output type	Input attribute type	Default conversions
String attribute formatter	Text string	Text string	Keep unchanged
		Number	Round to a certain number of significant or decimal digits, remove values higher or lower than a certain threshold
Number formatter	Number	Text string	Attempt to parse the string
		Number	Keep unchanged
Colour formatter	Colour	Text string	Convert from a CSS‐style string (e.g., “red,” “#FF0000,” or “rgb(255, 0, 0)”)
		Number	Map the number to a colour on a gradient

### 2.4 Module development

New modules can be developed to extend the capabilities of TreeViewer. The TreeViewer documentation contains information about the internal API of the program, in order for developers to access and extend key features. For example, a new module could be developed to add support for a new tree file format or to add a particular graphical element to the plot. Modules can be shared with the community by creating a “pull request” on the TreeViewer GitHub repository.

Software developers can leverage the lightweight development environment integrated within TreeViewer to help create new modules. Given basic knowledge of the C# programming language, the real‐time error correction and intelligent code completion features implemented through the CSharpEditor control (Bianchini, 2023b) make it possible to code new modules efficiently. Breakpoints can be used to pause execution of the module at critical points, in order to inspect the flow of the program and the value of local variables.

The recommended workflow when developing a new module for TreeViewer involves three steps. First, users are encouraged to use the *Custom script* modules to write an initial version of their source code. This allows quick prototyping of the source code, because the code is compiled and executed by TreeViewer at runtime, and fixing bugs or making changes does not require the program to be restarted. After an initial version of the new module has been developed, the second step is to use the “Module creator” function of TreeViewer to produce a full module prototype and test it. The “Module creator” supports the same code‐editing features as the custom script editors, but it makes it possible to create full‐fledged modules that are loaded by TreeViewer at startup. Thus, making changes to the code at this stage requires restarting the program, which is why we recommend polishing the code using the *Custom script* modules first. In the final step, module developers identify parameters used in the source code that should be modifiable by the user. These parameters should be exposed in the TreeViewer UI as described in the TreeViewer developer's guide (Bianchini, 2021c), and a brief description for each parameter and for the module should be provided in the documentation comments; this will allow TreeViewer to automatically generate a manual for the new module.

### 2.5 Digital signatures

The digital signature for a TreeViewer module is an RSA‐encrypted hash (Rivest et al., 1978) of the source code of the module, created and verified by TreeViewer using the SHA‐512 hashing algorithm (Dang, 2015) and the RSASSA‐PKCS1‐v1_5 signature scheme (Jonsson & Kaliski, 2003). Briefly, this involves a key pair consisting of a “private” key and a “public” key. The private key is available only to the user who generated it, while the public key is distributed to other users. The signature is then created by computing a hash digest of the source code, which is then encrypted using the private key. Validating the signature involves decrypting it using the public key and comparing the resulting value to a newly computed hash of the source code. The two hashes will match only if the public key corresponds to the private key used for encryption and if the source code has not been altered. Therefore, both the identity of the author or distributor of the source code and the integrity of the source code are guaranteed (Rivest et al., 1978).

Public keys for modules released through the TreeViewer GitHub repository are included in the source code of the program; a user attempting to install a module with an invalid signature will be presented with a warning message and will have the option to install the module anyways or to abort. An invalid signature may occur either because the module has been signed using a private key, whose public key counterpart is not included in the TreeViewer source code, or because the source code has been (possibly maliciously) altered. Users are encouraged not to install modules with an invalid signature unless they understand why the signature check failed and trust the source of the module. TreeViewer has a command‐line option to generate an RSA key pair. Combined with the open‐source nature of the software, this potentially enables other developers to make changes to the set of trusted public keys and create versions (“forks”) of TreeViewer allowing the installation of modules from alternative repositories.

Tree files containing source code are signed in a similar way. When the program is executed for the first time, a public/private key pair for the user is generated. The private key is then used to sign tree files, while the public key is also included in the file. The first time that user A opens a file that has been created by user B, a warning message is displayed, explaining that the tree file contains source code that could potentially be malicious. User A has then three options: the first option is to open the tree file discarding all the source code, thus reverting attribute formatters to the default settings; this may change the appearance of the plot. The second option is to allow loading the source code just once, while the final option is to “trust” the author of the file. This last option should be selected only when there is trust in both the author of the file and the integrity of the communication channel over which the file has been received (e.g., that there has not been a man‐in‐the‐middle or similar attack). In this case, the public key contained in the tree file will be stored in the user's cache, and if user A opens other files coming from user B, source code contained therein will automatically be trusted. This allows collaboration within groups without dialog boxes showing up every time a file is opened, while still allowing a certain degree of security for files coming externally. It should be noted that the signature can be used to assess the integrity of a file and the identity of its author only after the author's public signature has been stored in TreeViewer's key cache. In principle, it would also be possible to create an online repository of public keys and associated identities (verified, e.g., through an academic email address).

## 3 RESULTS

Here, we present an example using TreeViewer to display age estimates on a time‐calibrated phylogenetic tree. Time‐calibrated trees can be obtained, for example, from a Bayesian molecular clock analysis using software such as MrBayes (Ronquist & Huelsenbeck, 2003), PhyloBayes (Lartillot et al., 2009), RevBayes (Höhna et al., 2016), and BEAST (Suchard et al., 2018). These can be used by TreeViewer to compute a consensus tree and to plot the age distribution for each node as a violin plot. Such plots visualise the posterior probabilities, and these are more informative than simple confidence interval bars; this is particularly useful in the case of asymmetric age distributions.

### 3.1 Consensus tree

The tree file at https://treeviewer.org/manual/clock/clock.tre (also available in the Supplementary Information S1), generated after running a Bayesian molecular clock analysis of Cyanobacteria in PhyloBayes v4.1e (Lartillot et al., 2009), contains 1000 trees, in which each tree contains 42 taxa. When the tree file is opened, TreeViewer recognises that it contains multiple trees and automatically computes a greedy consensus tree (Bryant, 2003). The options of the “Consensus” *Transformer* module can be used to change how the consensus tree is computed (e.g., adding a burn‐in or sampling a subset of the trees), but in this case the default values are adequate.

### 3.2 Initial tree plot

**FIGURE 6:**
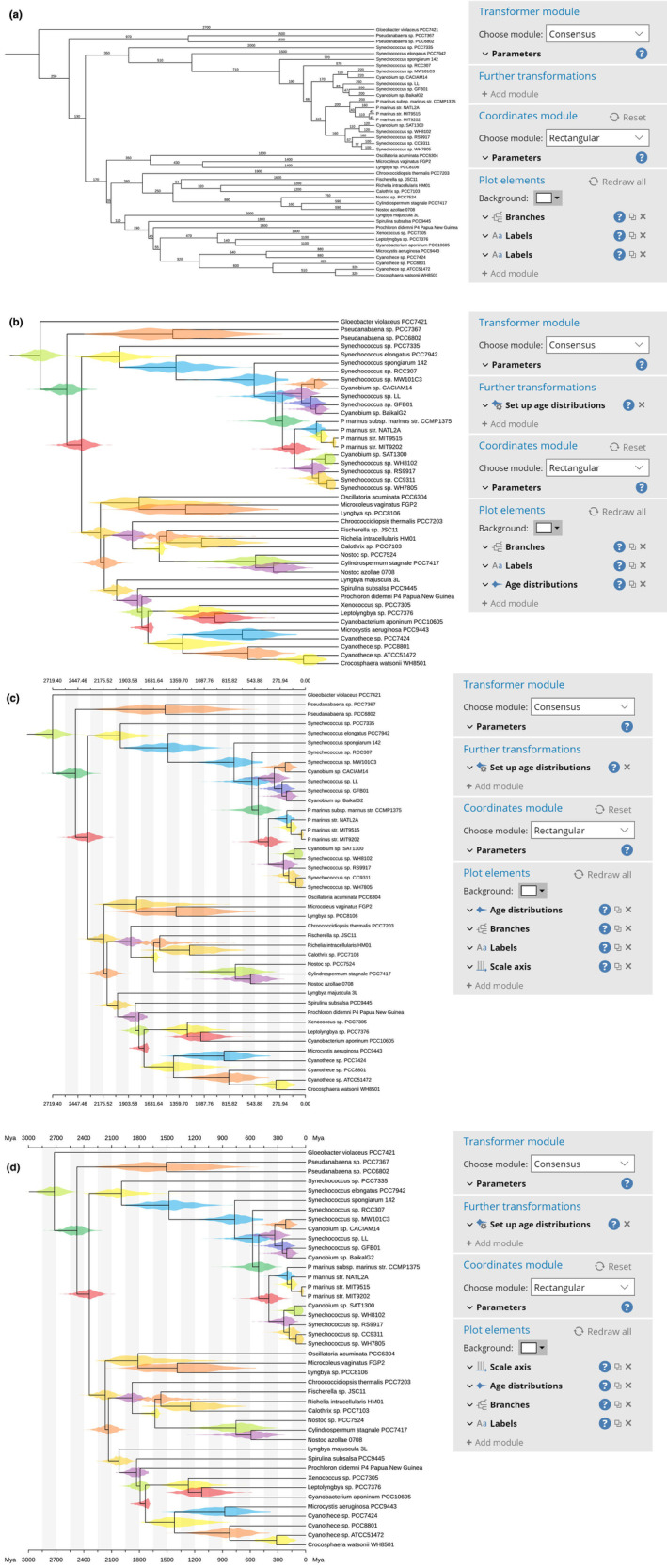
Steps performed in this example. (a) The file containing the trees has been opened in TreeViewer, which computed the consensus tree and displayed it. (b) Age distributions have been computed and plotted on the tree. (c) A scale axis has been added to the plot. (d) All the module parameters have been fine‐tuned in order to obtain a publication‐ready figure (shown enlarged in Figure 7).

The tree is shown automatically as a rooted tree (Figure 6a), and three *Plot action* modules are enabled: the “Branches” module, which draws the tree branches, and two instances of the “Labels” module. The first “Labels” module draws the taxon names at the tips of the tree, while the second “Labels” module draws the branch length over each branch. To make the plot easier to view, the second instance of the “Labels” module can be removed. Furthermore, to make the tree more compact, the “Width” parameter of the “Rectangular” *Coordinates* module can be reduced to 500.

### 3.3 Age distributions

Plotting the age distribution for each node requires a two‐step process: first, the age distributions need to be computed by using the “Set up age distributions” *Further transformation* module. By default, this module will also associate the mean age and 89% highest density interval (McElreath, 2020) to each node, which can be used, for example, to draw node bars showing the confidence interval (the default 89% threshold can be changed). To draw the age distributions, the “Age distributions” *Plot action* module needs to be enabled. This will draw a violin plot at each node in the tree, showing the posterior age distribution estimate for the node (Figure 6b). By default, each violin plot is drawn using a randomly chosen colour, but this can be changed by disabling the “Auto colour by node” option in the module parameters.

In TreeViewer, the plot elements are drawn in the order they are displayed in the left panel, from top to bottom; this means that the newly added age distributions are drawn above the tree branches and labels. To avoid obscuring the tree topology, the “Age distributions” module can be dragged up, so that it is the first module in the plot element list. Furthermore, some of the age distribution plots overlap, due to the limited available vertical space. The vertical spacing of the tips in the tree can be increased by opening the options for the *Coordinates* module and setting the “Height” to a higher value (e.g., 800).

### 3.4 Age axis

Additionally, it is possible to add an age axis to the plot, so that the age of the various groups can be read from the tree. This can be done by enabling the “Scale axis” *Plot action* module (Figure 6c). By default, the spacing between consecutive “ticks” on the axis drawn by this module is computed automatically. To make the axis more pleasant, the “Tick spacing” can be increased to 150, the “End” to 3000, and the “Digits” can be set to 0. In this tree, node ages are expressed in units of millions of years; to show this on the plot, the value of the “Units” parameter can be set as “Mya,” which will show the unit at the start and end of the scale axis. The module can be moved up in the list of plot elements, so that the scale axis is drawn before all the other layers in the plot. Finally, the font size of the labels and of the scale axis can be increased by clicking on the respective “Font” buttons and entering “12” in the “Font size” box (Figure 6d).

### 3.5 Exporting the plot

**FIGURE 7:**
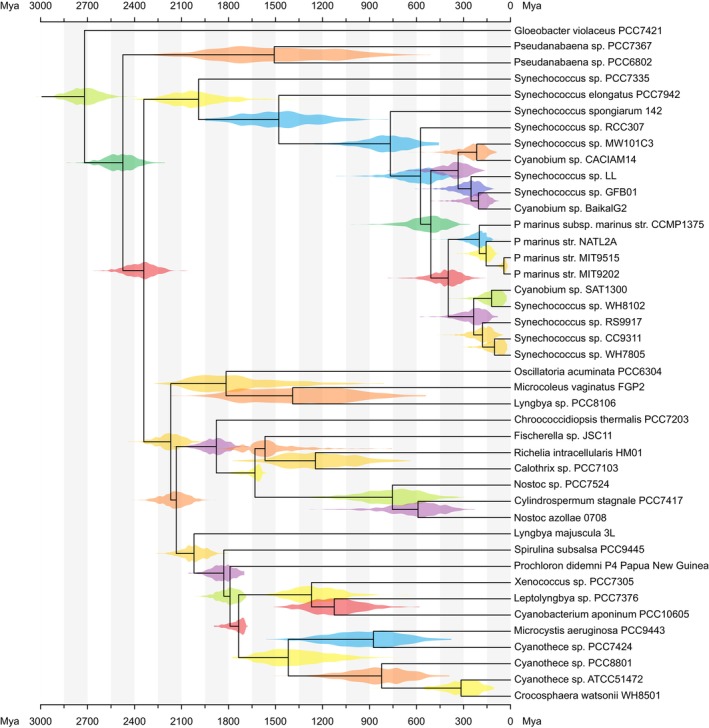
Tree produced following the steps in this example. For each node, a violin plot shows the posterior age distribution estimate for the node. Ages in the scale axis are expressed as millions of years since the present day.

A finished figure looks similar to Figure 7. The plot can be exported in PDF, SVG, PNG, or TIFF format, and the tree file can be saved in a format (e.g., NEXUS or binary) that preserves information about the enabled modules. When such a file (available at https://treeviewer.org/manual/clock/clock_finished.tbi and in the Supplementary Information S2) is opened, TreeViewer will automatically replicate all the processing steps involved in going from the initial tree file to the finished plot. Furthermore, the “Apply to other tree” button in the “Edit” tab can be used to apply the current style to a different file (naturally, some fine‐tuning might be necessary). For example, in a Bayesian analysis, this would make it possible to create an initial draft of the figure with tree samples from a chain that has not converged; then, once the analysis has run for long enough, the plot style can be applied to the final sample of trees with just a few clicks.

A more detailed version of this example, showing some additional features of TreeViewer, can be found in the TreeViewer online manual (Bianchini, 2021d). Furthermore, TreeViewer can plot multiple different age distributions on the same tree; this is useful, for example, when comparing fossil age calibrations, the effective prior distribution, and the posterior distribution. A tutorial on how to do this is also available in the TreeViewer online manual (Bianchini, 2023c). Many more examples and tutorials showcasing the capabilities of TreeViewer are available in the online manual (Bianchini, 2021e).

## 4 DISCUSSION

Many software tools exist to plot and visualise phylogenetic trees, which can be broadly categorised into interactive software with a GUI and software libraries for use through a scripting/programming language (some representative examples are shown in Table 3). The Interactive Tree Of Life (iTOL; Letunic & Bork, 2021), for instance, is an online GUI tool that enables users to plot, annotate, and manage phylogenetic trees; FigTree (Rambaut, 2018) is a simple desktop application to draw phylogenetic trees, including some capabilities for annotation and manipulation. Examples of software libraries include ggtree (Yu et al., 2017), an R package, and the Environment for Tree Exploration (ETE; Huerta‐Cepas et al., 2016), a Python package. TreeViewer aims to bridge the gap between the two categories, providing users with both the ease of use of a GUI software and powerful scripting capabilities.

**TABLE 3:** Overview of phylogenetic tree visualisation software. For each program, the table shows whether it is free and open source and the licence under which it is released, the operating system (OS) compatibility (here, “All” refers to Windows, Linux and macOS), whether it provides a GUI and/or a command‐line interface (CLI), and the programming language in which it was created.

Software	Free and open source	Licence	OS compatibility	Interface	Programming language
TreeViewer	Yes	AGPL‐3.0	All	GUI, CLI	C#
iTOL	No	Proprietary	All (online)	GUI, (CLI^a^)	JavaScript^b^
FigTree	Yes	GPL‐2.0	All	GUI	Java
Ggtree	Yes	Artistic‐2.0	All	CLI	R
ETE	Yes	GPL‐3.0	Linux and macOS	CLI, (GUI^c^)	Python

^a^ In addition to the main GUI, iTOL allows users with an active subscription to upload and export trees from the command line.

^b^ The latest versions of the client‐side visualisation engine for iTOL are written in JavaScript (Letunic & Bork, 2021); source code for the server‐side components is not freely available, but the original release used Perl scripts (Letunic & Bork, 2007).

^c^ In addition to the main Python command‐line interface, ETE has basic interactive capabilities.

One of the main attractions of the iTOL (Letunic & Bork, 2021) is its cloud‐based nature, since users do not need to download any software on the machine, and trees saved on their online workspace are available from any workstation. The iTOL portal also allows users to share annotated trees with other collaborators and to create figures for publication. However, unrestricted use of the software requires a paid subscription (Letunic & Bork, 2021), and the source code for the software is not publicly available. While installing iTOL on a local machine is possible, it requires liaising with the software creators.

FigTree (Rambaut, 2018) is a Java‐based program that was originally designed to display phylogenetic trees produced by BEAST (Suchard et al., 2018). It is very accessible, and it can be easily used to perform basic annotations on the trees (e.g., adding symbols at the tips or internal nodes, changing the display colour of part of the tree, and displaying scale bars). There are, however, some limitations to the number and the kind of annotations that can be added to the tree. Trees produced often need to be exported and edited using other software (e.g., Adobe Illustrator or Inkscape) to prepare them for publication. FigTree is open source (released under a GNU GPLv2 licence), and it is available for Windows, Linux, and macOS.

The ggtree R package (Yu et al., 2017) allows users to plot phylogenetic trees using a “layered” syntax derived from the general‐purpose ggplot2 (Wickham, 2016) R graphics package. In this way, a basic tree geometry can be extended with many layers of additional elements such as labels, symbols, and heatmaps. Since all of this happens within an R scripting environment, users can manipulate the trees using other popular packages such as ape (Paradis et al., 2004) or phytools (Revell, 2012). The ggtree package is open source (released under the Artistic License 2.0) and available for Windows, Linux, and macOS.

The ETE Python package (Huerta‐Cepas et al., 2016) provides similar capabilities for Python users. In addition to tree drawing and annotation capabilities, ETE can perform some evolutionary analyses and can access data from online databases such as the NCBI taxonomy (Schoch et al., 2020) and the Genome Taxonomy Database (Parks et al., 2022). Furthermore, it provides some interactive features. ETE is open source (released under a GNU GPLv3 licence), but does not support Windows, and can only be installed on Linux and macOS. A limited online version allowing users to plot a tree together with a sequence alignment is also available.

Code that uses software libraries such as ggtree and ETE can be easily incorporated into analysis pipelines, and the scripts used to produce the plots can be used to replicate the plot or adapt it to a different dataset with minimal effort. However, one of their drawbacks is that users are required to be familiar with the relevant programming language, unlike GUI tools such as iTOL and FigTree. As a result, GUI tools appeal to a much wider audience. For instance, at the time of writing, five publications describing ggtree (Xu et al., 2022; Yu, 2020, 2022; Yu et al., 2017, 2018) have a total of 1862 citations, and two publications describing ETE (Huerta‐Cepas et al., 2010, 2016) have a total of 1685 citations. In contrast, five publications describing iTOL (Letunic & Bork, 2007, 2011, 2016, 2019, 2021) have a total of 15,388 citations. FigTree is not associated with an official publication, which makes tracking its use more difficult; however, a Google Scholar search suggests upwards of 7700 citations.

TreeViewer has a GUI interface that can be used to produce simple tree plots without much effort (similar to iTOL or FigTree), though the creation of complex, multi‐annotation plots requires some familiarity with the program. The “Apply to other tree” option reuses a plot style for a different tree; this is similar to changing the input file in an R or Python script, but everything happens through the TreeViewer GUI. The command‐line interface can be used to integrate the software into pipelines and to reproduce the same plot after minor changes. At the same time, the integrated C# scripting environment allows advanced users to manipulate the tree without restriction (like ggtree or ETE). The free and open‐source licensing scheme and cross‐platform compatibility allow users to run TreeViewer with full capabilities on almost any machine (unlike ETE) and without having to pay any fees (unlike iTOL).

## 5 CONCLUSION

TreeViewer provides new and versatile capabilities for drawing, annotating, and analysing phylogenetic trees. At present, trees can be annotated with discrete and continuous character data, sequence alignments, age distributions, images, highlights, and stochastic mapping results. The modular design allows both the main software developers and experienced users to expand TreeViewer's capabilities without having to make significant alterations to the main code base.

The user‐friendly interface and intuitive zoom‐and‐drag functionalities allow inexperienced users to quickly produce a plot and explore the relationships between the taxa included in the tree. Detailed documentation and tutorials are available for users to learn about the capabilities of the program and how to use and combine them. Furthermore, every module has its own manual, accessible from within TreeViewer's user interface. Using the lightweight integrated development environment and the information in the developer's guide (included in the online manual), advanced users can create small scripts and/or new modules to change the structure of the tree or the way in which it is displayed.

TreeViewer is freely available for Windows, Linux, and macOS (both Intel‐based and Apple Silicon‐based) computers. The source code and compiled executable files can be downloaded from the program's website at https://treeviewer.org, which also contains links to the documentation, tutorials, and GitHub repository (https://github.com/arklumpus/TreeViewer), where users can submit feedback, request help, and/or report bugs.

## AUTHOR CONTRIBUTIONS

**Giorgio Bianchini:** Conceptualization (lead); methodology (lead); software (lead); validation (lead); visualization (lead); writing – original draft (lead); writing – review and editing (equal). **Patricia Sánchez‐Baracaldo:** Funding acquisition (lead); supervision (lead); writing – original draft (supporting); writing – review and editing (equal).

## FUNDING INFORMATION

Funding support for this work came from a University of Bristol Scholarship to GB and a Royal Society University Research Fellowship to PS‐B. For the purpose of open access, the authors have applied a Creative Commons Attribution (CC BY) licence to any author‐accepted manuscript version arising from this submission.

## CONFLICT OF INTEREST STATEMENT

The authors declare that they have no competing interests.

## Supporting information


Data S1

Data S2

## Data Availability

The source code for TreeViewer and all associated example files and tutorials are available from the TreeViewer GitHub repository at https://github.com/arklumpus/TreeViewer and archived on Zenodo at https://zenodo.org/doi/10.5281/zenodo.7768343.
